# Renal Replacement Therapy: Purifying Efficiency of Automated Peritoneal Dialysis in Diabetic *versus* Non-Diabetic Patients

**DOI:** 10.3390/jcm4071518

**Published:** 2015-07-22

**Authors:** Nicanor Vega-Diaz, Fayna Gonzalez-Cabrera, Silvia Marrero-Robayna, Raquel Santana-Estupiñan, Roberto Gallego-Samper, Fernando Henriquez-Palop, Patricia Perez-Borges, José Carlos Rodriguez-Perez

**Affiliations:** Nephrology Service, Hospital Universitario de Gran Canaria Dr. Negrin, Universidad de Las Palmas de Gran Canaria, 35019 Las Palmas de Gran Canaria, Spain; E-Mails: fayna_gc@hotmail.com (F.G.-C.); silvi.mr84@hotmail.com (S.M.-R.); raquelsestupinan@hotmail.com (R.S.-E.); rgalsam@gobiernodecanarias.org (R.G.-S.); fhpalop@gmail.com (F.H.-P.); pperbor@gmail.com (P.P.-B.); jrodperd@gobiernodecanarias.org (J.C.R.-P.)

**Keywords:** diabetes, automatic peritoneal dialysis, adequacy, water balance, sodium balance

## Abstract

Background: In order to reduce the cardiovascular risk, morbidity and mortality of peritoneal dialysis (PD), a minimal level of small-solute clearances as well as a sodium and water balance are needed. The peritoneal dialysis solutions used in combination have reduced the complications and allow for a long-time function of the peritoneal membrane, and the preservation of residual renal function (RRF) in patients on peritoneal dialysis (PD) is crucial for the maintenance of life quality and long-term survival. This retrospective cohort study reviews our experience in automatic peritoneal dialysis (APD) patients, with end-stage renal disease (ESRD) secondary to diabetic nephropathy (DN) in comparison to non-diabetic nephropathy (NDN), using different PD solutions in combination. Design: Fifty-two patients, 29 diabetic and 23 non-diabetic, were included. The follow-up period was 24 months, thus serving as their own control. Results: The fraction of renal urea clearance (Kt) relative to distribution volume (V) (or total body water) (Kt/V), or creatinine clearance relative to the total Kt/V or creatinine clearance (CrCl) decreases according to loss of RRF. The loss of the slope of RRF is more pronounced in DN than in NDN patients, especially at baseline time interval to 12 months (loss of 0.29 mL/month *vs.* 0.13 mL/month, respectively), and is attenuated in the range from 12 to 24 months (loss of 0.13 mL/month *vs.* 0.09 mL/month, respectively). Diabetic patients also experienced a greater decrease in urine output compared to non-diabetic, starting from a higher baseline urine output. The net water balance was adequate in both groups during the follow up period. Regarding the balance sodium, no inter-group differences in sodium excretion over follow up period was observed. In addition, the removal of sodium in the urine output decreases with loss of renal function. The average concentration of glucose increase in the cycler in both groups (DN: baseline 1.44 ± 0.22, 12 months 1.63 ± 0.39, 24 months 1.73 ± 0.47; NDN: baseline 1.59 ± 0.40, 12 months 1.76 ± 0.47, 24 months 1.80 ± 0.46), in order to maintain the net water balance. The daytime dwell contribution, the fraction of day and the renal fraction of studies parameters provide sustained benefit in the follow-up time, above 30%. Conclusions: The wet day and residual renal function are determinants in the achievement of the objective dose of dialysis, as well as in the water and sodium balance. The cause of chronic kidney disease (CKD) does not seem to influence the cleansing effectiveness of the technique.

## 1. Introduction

The incidence and prevalence of diabetes mellitus (DM) is estimated to continue increasing worldwide. In the year 2014, 387 million people had diabetes (prevalence 8.3%) according to the International Diabetes Federation Atlas [1]. In the USA in 2012, Diabetes Mellitus was the most important cause of end-stage renal disease (ESRD), with an incidence of 50.53% and a prevalence of 38.16% (731 per million population, pmp) [2]. In Spain, the incidence of diabetics who started Renal Replacement Therapy (RRT) in 2012 was 24.91% and specifically in the Canary Islands was 43.75%. Meanwhile, the prevalence was 14.6% (170.4 pmp) and 39.1% (454.1 pmp) respectively [3].

The role of peritoneal dialysis (PD) in renal replacement therapy in patients with diabetic ESRD is well established and used world-wide [4,5,6].

The use of dextrose-containing solutions in peritoneal dialysis (PD) is thought to be associated with glucose-related toxicity both, at systemic level and also direct to the peritoneal membrane. The changes in peritoneal membrane thickness and vascular alterations in relation to the duration of dialysis are caused mainly by glucose and glucose degradation products, such as advanced glycation end-products (AGEs). Therefore, there has been a considerable interest in minimizing the use of dextrose exposure during PD. The peritoneal dialysis solutions used in combination have reduced the complications and allows a long-time function of the peritoneal membrane, as they also can contribute to reducing the cardiovascular risk of PD patients [7,8,9]. On the other-hand, preservation of residual renal function (RRF) in patients on PD is crucial for the maintenance of life quality and long-term survival [10,11,12,13,14].

This retrospective cohort study reviews our experience of automatic peritoneal dialysis (APD) patients in a single center on the Canary Islands, with ESRD secondary to diabetic nephropathy (DN) in comparison to non-Diabetic nephropathy (NDN), using different PD solutions in combination. Two daily exchanges; first icodextrin or glucose and second aminoacids, were made, trying to minimize the dextrose use in the APD nocturnal period. In addition, we evaluated the contribution of diurnal period to overall peritoneal doses of dialysis and the residual renal function contribution to overall doses of dialysis during a two year follow-up period.

## 2. Materials and Methods

### 2.1. Patient Characteristics and Selection Criteria

The total patient number consisted of 52 ESRD patients, 32 men and 20 women, with a mean age of 60.38 ± 13.47 (range, 26–82) years. The selection criteria were that patient age is greater than 18 years, and the use of similar cycler program schedules at home in order to reach the objectives of appropriate dialysis dose. This is considered to be the sum of clearances contributed by the cycler, by the diurnal period, and by residual renal function.

### 2.2. Peritoneal Membrane Permeability

The peritoneal permeability study was performed annually, as a routine. Furthermore, the baseline study was performed at the end of the training period. To realize a qualitative analysis of the peritoneal permeability, Peritoneal equilibrium test (PET) was used according to the standardized and described methodology by Twardowski *et al.* [15].

### 2.3. APD Protocol

None of the patients was prescribed a “dry day”; that is, all patients used dialysis fluid during the day. The initial dialysis prescription at inclusion was adjusted according to the theoretical requirements of the patient’s dialysis dose and residual renal function. The infusion volume during the nocturnal treatment was adjusted according to the patient’s weight and ability to tolerate this volume. The total diurnal volume was 4 l in two 2 l dwells, one of which was manually performed around noon or during afternoon hours, and usually consisted of a 1.1% amino acid PD solution.

An outline of baseline standard cycler therapy is: continuous cycling peritoneal dialysis (CCPD) modality, time 9 h, total volume at night 11,500 mL, infusion volume 2300 mL, number of cycles 5, dwell time 75 min, last dextrose infusion different (icodextrin) or glucose mixture 2000 mL. If the patient complained of successive controls discomfort or abdominal pain during infusion or drainage, tidal 85% modality was programmed for therapy at home. Loop diuretics (one or both of furosemide and torasemide) are prescribed to maintain the urine output.

For the first study of dialysis dose quantification after one month of therapy, a night stay at the hospital dialysis unit was scheduled. The patient collected the drained dialysate in the preceding manual exchange as well as a 24-h urine collection. The first cycler drainage was separately collected and mixed with the dialysate, which the patient brought from home. Blood samples were taken after the patient’s connection and disconnection from the cycler, and dialysate samples were taken from the total diurnal and nocturnal drainage for analytical measurements.

### 2.4. Total Dialysis Dose and that Contributed by the Cycler

Diurnal and nocturnal period dialysis doses were measured separately. Total clearance was calculated as the sum of the renal and peritoneal clearances. Urea Kt/V index (urea clearance (Kt) relative to distribution volume (V) (or total body water)) and creatinine clearance (CrCl) were calculated according to Keshaviah *et al.* [16] and Twardowski [17]. Body surface (BS) was estimated according to Du Bois and Du Bois [18] and total body water (TBW) according to Watson *et al.* [19]. RRF was evaluated as (Urea clearance + Creatinine clearance)/2.

(1) $$PC_{W}\;=\;\frac{V_{D}xS_{D}}{S_{P}}x\;7\;(\text{L})$$

(2) $$P\frac{Kt}{V}\;=\frac{PC_{W}Urea}{TBW}$$

(3) $$PCrC_{W}=\;\frac{PC_{W}Creatinine\;x\;1.73}{BS}$$

PC: peritoneal clearance; W: week; V: volume; D: drain; S: solute; P: plasma; Cr: creatinine; BS: body surface.

### 2.5. Ultrafiltration Net (UF Net), Water Balance and Sodium Removal

UF Net is the sum of UF in the night and the UF in the day. The Water Balance is the sum of UF Net plus the residual urine output. Peritoneal sodium removal (TMNa) was estimated according to Nolph [20]. Peritoneal sodium balance is the sum of peritoneal sodium removal in the night and peritoneal sodium removal in the day. Sodium removal is the sum of urinary excretion and peritoneal balance.

(4) $$TMNa\;(mEq)=\left(V_{D}xD_{Na}\right)-\;(V_{I}xI_{Na})$$

I: infusion; D: drain.

The creatinine, urea, glucose and sodium parameters, both in plasma and dialysate, were measured with Modular Analytics (PPE) (Roche Diagnostics, Basel, Switzerland) equipment.

### 2.6. Statistical Methods

Normally distributed variables were expressed as mean ± standard deviation (SD) unless otherwise noted. Statistical significance was set at the level of *p* < 0.05. As we were dealing with a longitudinal study, where patients were their own controls at three different times, basal, one and two year, differences between the different times were analyzed with the general linear model of repeated samples (GLM Repeated Measures) (variance analysis). Using this procedure, null hypothesis may be contrasted on the effects of both the intersubject and the intrasubject factors. It allows investigation of the interactions between the factors and also the individual effects of such factors. The effects of steady covariables and the interactions of the covariables with the intersubject factors can also be included. This procedure also allows univariate and multivariate analyses. Type III square sum method is used in multivariate analysis.

After verifying that the continuous variables, absolute value and those in the groups, are set to normal (Kolmogorov-Smirnov), for intergroup comparison at all time (Basal, 12 months and 24 months) we used parametric tests (Student’s *t*-test). Moreover, in univariate analysis we used chi-square test.

All statistical analyses were performed with SPSS statistical software (Version 17.0; SPSS Inc., Chicago, IL, USA).

## 3. Results

The main characteristics of both groups, DN and NDN patients, are shown in Table 1. Basal peritoneal transport estimated by creatinine in PET showed that four diabetic patients had inherent high transport starting therapies, a year later they had lost this feature. In any case, the high permeability transport was not acquired in the two year follow-up. Forty patients using icodextrin and 28 patients did not suffer any episode of peritoneal infection during the study period. No association was observed between underlying disease *vs.* gender (χ^2^ = 0.24, *p* = 0.61), *vs.* peritoneal permeability grouped (χ^2^ = 0.94, *p* = 0.46). Neither was it observed with icodextrin (χ^2^ = 0.24, *p* = 0.76), or peritoneal infection (χ^2^ = 2.15, *p* = 0.14).

**Table 1:** Characteristics of diabetic and non-diabetic patients.

		Diabetics	Non Diabetics	Overall
*N*		29	23	52
Age (years)		59.28 ± 11.71 (33–72)	61.78 ± 15.56 (26–82)	60.38 ± 13.47 (26–82)
Gender	Men	17	15	32
	Women	12	8	20
Peritoneal Transport (PT) Creatinine (Cr) Basal PET	High	4	1	5
	High Average	9	7	16
	Low Average	7	8	15
	Low	9	7	16
PT Cr Grouped	High + High Average	13	8	21
	Low Average + Low	16	15	31
Icodextrin	Yes	21	19	40
	No	8	4	12
Peritonitis	Yes	16	8	24
	No	13	15	28

PET: peritoneal equilibrium test.

In general, patients led to weight gain in the first year. However, while this gain is significant in NDN patients with significant increase in body mass index (BMI); in diabetic patients it was not significant.

However, the increase in TBW was significant in both groups (Table 2). No differences were observed, between DN and NDN, at the two year follow-up.

**Table 2:** Weight and anthropometric parameter.

**Diabetics**	**Baseline (a)**	**12 months (b)**	***p* (a *vs.* b)**	**24 months (c)**	***p* (a *vs.* c)**
Weight (kg) (1)	71.90 ± 12.50	73.88 ± 13.95	0.080	74.23 ± 13.50	0.075
BS (m^2^) (2)	1.78 ± 0.16	1.80 ± 0.16	0.05	1.81 ± 0.16	0.05
BMI (kg/m^2^) (3)	26.80 ± 4.97	27.55 ± 5.58	0.086	27.67 ± 5.60	0.076
TBW (l) (4)	36.46±5.11	37.07±5.22	0.047	37.06±4.87	0.105
**Non Diabetics**	**Baseline (a)**	**12 months (b)**	***p* (a *vs.* b)**	**24 months (c)**	***p* (a *vs.* c)**
Weight (kg) (5)	76.95 ± 15.69	82.24 ± 16.52	0.001	82.87 ± 10.03	0.001
BS (m^2^) (6)	1.85 ± 0.20	1.90 ± 0.21	0.001	1.90 ± 0.22	0.001
BMI (kg/m^2^) (7)	28.20 ± 5.36	30.11 ± 5.16	0.001	30.27 ± 5.44	0.001
TBW (l) (8)	38.75 ± 7.23	40.24 ± 7.67	0.001	40.37 ± 8.11	0.001

BS: body surface; BMI: body mass index; TBW: total body water.

At baseline, residual renal function (RRF) is significantly higher in DN patients compared to NDN (*p* = 0.019) and creatinine is significantly higher in NDN patients compared to DN (*p* = 0.007). As expected, in both groups over time, serum creatinine increases with loss of renal function and it is significantly higher in NDN patients compared to DN at 12 (*p* = 0.037) and 24 months (*p* = 0.033) respectively. No differences between groups, in the matter of urea and serum sodium levels, were found. At the time of the two year follow-up, serum sodium levels are slight, but tangibly lower in diabetic patients from baseline (Table 3).

**Table 3:** Serum biochemical parameters and Residual Renal Function (RRF).

**Diabetics**	**Baseline (a)**	**12 months (b)**	***p* (a *vs.* b)**	**24 months (c)**	***p* (a *vs.* c)**
Urea (mg/dL) (1)	109.34 ± 27.50	114.05 ± 31.34	0.350	109.88 ± 26.68	0.907
Cr (mg/dL)(2)	4.48 ± 1.63	5.81 ± 2.12	0.001	6.27 ± 2.01	0.001
Na (mmol/L) (3)	138.62 ± 2.39	137.83 ± 2.53	0.176	137.31 ± 2.67	0.037
RRF (mL/min) (4)	8.43 ± 4.00	4.92 ± 3.72	0.002	3.31 ± 3.09	0.001
**Non-Diabetics**	**Baseline (a)**	**12 months (b)**	***p* (a *vs.* b)**	**24 months (c)**	***p* (a *vs.* c)**
Urea (mg/dL) (5)	118.61 ± 23.95	121.85 ± 25.42	0.536	117.07 ± 27.98	0.784
Cr (mg/dL) (6)	5.86 ± 1.91	7.12 ± 2.42	0.001	7.54 ± 2.43	0.001
Na (mmol/L) (7)	137.76 ± 2.55	137.35 ± 2.55	0.865	137.63 ± 2.43	0.863
RRF (mL/min) (8)	5.96 ± 3.15	4.37 ± 4.43	0.001	3.12 ± 3.63	0.004

Cr, creatinine; Na, sodium; RRF, residual renal function; 4b *vs.* 4c, *p* = 0.011.

The percentage of RRF mean loss was: DN patients, between baseline and 12 months lost 41.63%, while between 12 and 24 months lost 32.72%. In the first two years, they had lost 60.7% of RRF. NDN patients, between baseline and 12 months lost 26.7% of the RRF, while between 12 and 24 months lost 28.6%, in the first two years, they had lost 47.7% of RRF.

**Figure 1:**
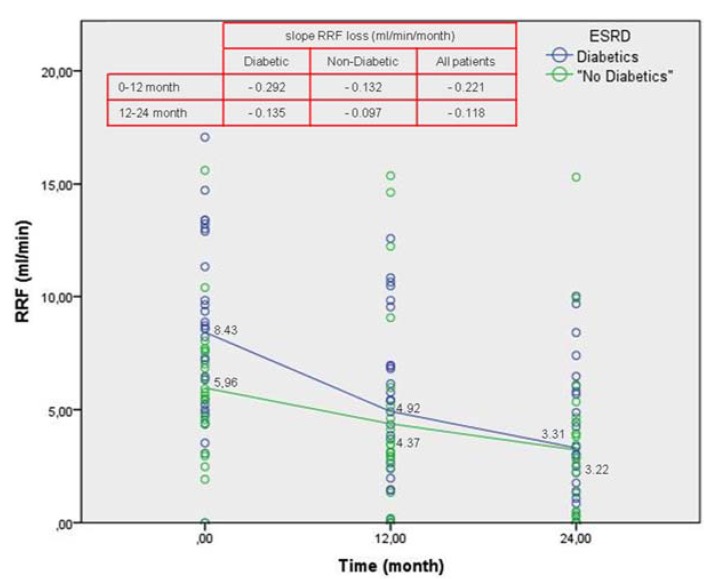
Residual Renal Function (RRF).

The slope of RRF loss is more pronounced over the first year in DN patients, and more attenuated in the second year. In NDN patients, RRF loss is more attenuated and progressive over the two years (Figure 1).

The weekly Kt/V, was higher in DN compared to NDN patients. The reason is that the fraction of Kt/V corresponding to peritoneal cycle time (PCt) and renal is significantly higher in DN patients at least at baseline and at 12 months, while no differences between groups in the peritoneal day time (PDt) (Table 4) were found. The biggest difference in the fraction corresponding to PCt, can be justified because the estimated TBW is lower in DN patients than in NDN. Meanwhile, the Kt/V RRF fraction can be justified for this reason with the addition that the RRF is higher in diabetics.

The more dialysate urea saturation, in both groups during the PDt, minimizes differences, due to a lower TBW in diabetics.

At baseline, Kt/V PCt (*p* = 0.034), peritoneal (*p* = 0.037), renal (*p* = 0.001) and total (*p* = 0.001) is significantly higher in DN patients compared to NDN. Over time, Kt/V PCt at 12 months is significantly higher in DN patients compared to NDN.

The percentage fraction Kt/V PDt to total peritoneal is similar in both groups and was maintained throughout the two years. While the fraction of renal Kt/V relative to total Kt/V decreases according to the loss of RRF, reaching an equal situation at the two year follow-up.

No differences were found in the fraction of weekly CrCl on the PCt, PDt and Peritoneal intergroup or intragroup over the two year follow-up. Significant differences were observed in the fraction corresponding to the RRF (*p* = 0.017) and the total peritoneal creatinine clearance (*p* = 0.004), which was greater in diabetic patients at baseline. However, this statistical significance was lost at 12 and 24 months (Table 5).

PDt Creatinine Clearance fraction relative to the total peritoneal creatinine clearance is similar in both groups and was maintained throughout the two years, while the fraction of renal creatinine clearance relative to the total creatinine clearance decreases according to loss of RRF becoming equal at the two years.

**Table 4:** Urea Kt/V Index.

**Diabetics**	**Baseline (a)**	**12 months (b)**	***p* (a *vs.* b)**	**24 months (c)**	***p* (a *vs.* c)**
PCt (1)	1.67 ± 0.46	1.40 ± 0.26	0.006	1.41 ± 0.32	0.002
PDt (2)	0.78 ± 0.20	0.77 ± 0.16	0.412	0.77 ± 0.16	0.567
Peritoneal (3)	2.45 ± 0.52	2.16 ± 0.38	0.006	2.18 ± 0.39	0.017
Renal (4)	1.42 ± 0.63	0.82 ± 0.60	0.001	0.56 ± 0.51	0.001
Total (5)	3.87 ± 0.79	2.99 ± 0.66	0.001	2.74 ± 0.51	0.001
% PDt/Peritoneal	31.84%	35.64%	-	35.32%	-
% Renal/Total	36.69%	27.42%	-	20.44%	-
**Non-Diabetics**	**Baseline (a)**	**12 months (b)**	***p* (a *vs.* b)**	**24 months (c)**	***p* (a *vs.* c)**
PCt (6)	1.41 ± 0.36	1.25 ± 0.24	0.128	1.25 ± 0.37	0.108
PDt (7)	0.76 ± 0.15	0.73 ± 0.16	0.203	0.70 ± 0.23	0.106
Peritoneal (8)	2.17 ± 0.41	1.98 ± 0.36	0.087	1.95 ± 0.59	0.072
Renal (9)	0.86 ± 0.44	0.67 ± 0.66	0.182	0.51 ± 0.51	0.003
Total (10)	3.03 ± 0.55	2.65 ± 0.72	0.046	2.46 ± 0.583	0.007
% PDt/Peritoneal	35.02%	38.02%	-	35.89%	-
% Renal/Total	28.38%	25.28%	-	20.73%	-

Urea Kt/V index: urea clearance (Kt) relative to distribution volume (V) (or total body water); PCt: peritoneal cycle time; PDt: peritoneal day time; Peritoneal = PCt + PDt.

**Table 5:** Creatinine clearance (L/week/1.73 m^2^).

**Diabetics**	**Baseline (a)**	**12 months (b)**	***p* (a *vs.* b)**	**24 months (c)**	***p* (a *vs.* c)**
PCt (1)	32.20 ± 8.45	30.86 ± 8.20	0.394	31.73 ± 8.68	0.192
PDt (2)	22.91 ± 6.46	22.03 ± 4.79	0.221	22.80 ± 4.37	0.891
Peritoneal (3)	55.10 ± 12.66	52.90 ± 11.55	0.284	54.53 ± 10.53	0.798
Renal (4)	113.36 ± 57.11	65.65 ± 50.40	0.001	43.90 ± 41.40	0.001
Total (5)	168.36 ± 54.47	118.55 ± 47.78	0.001	98.43 ± 37.66	0.001
% PDt/Peritoneal	41.57%	41.64%	-	41.81%	-
% Renal/Total	67.33%	55.38%	-	47.50%	-
**Non-Diabetics**	**Baseline (a)**	**12 months (b)**	***p* (a *vs.* b)**	**24 months (c)**	***p* (a *vs.* c)**
PCt (6)	28.56 ± 10.82	30.08 ± 7.43	0.467	30.79 ± 6.39	0.282
PDt (7)	22.25 ± 6.77	21.31 ± 3.84	0.483	21.91 ± 4.48	0.188
Peritoneal (8)	48.84 ± 15.43	51.40 ± 9.80	0.415	52.10 ± 10.12	0.184
Renal (9)	79.28 ± 36.84	55.92 ± 56.01	0.027	40.97 ± 49.75	0.002
Total (10)	128.12 ± 38.49	107.32 ± 56.52	0.098	93.68 ± 56.75	0.009
% PDt/Peritoneal	45.56%	41.46%	-	42.05%	-
% Renal/Total	61.88%	52.11%	-	43.73%	-

PCt: peritoneal cycle time; PDt: peritoneal day time; peritoneal = PCt + PDt.

Regarding the peritoneal ultrafiltration (Table 6), there was no intergroup differences over the two years. Intragroup there is no differences in the basal UF (ultrafiltration) PCt (peritoneal cycle time) at 12 and 24 months, and is associated with increased average glucose concentration cycler (g/L) at all times, in both groups (DN: baseline 1.44 ± 0.22, 12 months 1.63 ± 0.39, 24 months 1.73 ± 0.47; NDN: baseline 1.59 ± 0.40, 12 months 1.76 ± 0.47, 24 months 1.80 ± 0.46).

**Table 6:** Ultrafiltration (UF) and water balance (WB) (mL/day).

**Diabetics**	**Baseline (a)**	**12 months (b)**	***p* (a *vs.* b)**	**24 months (c)**	***p* (a *vs.* c)**
UF PCt (1)	306.55 ± 453.77	634.00 ± 521.70	0.008	628.21 ± 555.11	0.016
UF PDt (2)	268.48 ± 411.75	261.34 ± 316.15	0.906	329.07 ± 408.51	0.458
Net UF (3)	575.03 ± 19.75	895.34 ± 628.78	0.004	957.28 ± 744.28	0.009
Diuresis (4)	1218.45 ± 495.01	779.31 ± 497.77	0.001	530.17 ± 432.77	0.001
WB (5)	1793.48 ± 689.60	1674.66 ± 725.58	0.040	1487.45 ± 824.59	0.048
% UF PDt/Net UF	46.64%	29.19%	-	34.38%	-
% Diuresis/WB	67.94%	46.54%	-	35.64%	-
**Non-Diabetics**	**Baseline (a)**	**12 months (b)**	***p* (a *vs.* b)**	**24 months (c)**	***p* (a *vs.* c)**
UF PCt (6)	426.65 ± 637.87	671.78 ± 507.54	0.040	825.26 ± 587.98	0.036
UF PDt (7)	349.09 ± 285.20	339.83 ± 307.96	0.906	371.70 ± 363.35	0.748
Net UF (8)	776.74 ± 703.52	1011.64 ± 654.32	0.135	1203.52 ± 788.97	0.064
Diuresis (9)	864.78 ± 483.86	730.43 ± 668.23	0.358	681.74 ± 540.64	0.124
WB (10)	1640.51 ± 792.37	1742.04 ± 990.25	0.646	1875.76 ± 840.83	0.298
% UF PDt /Net UF	44.94%	33.59%	-	30.88%	-
% Diuresis/WB	52.71%	41.93%	-	36.34%	-

PCt: peritoneal cycle time; PDt: peritoneal day time; UF: ultrafiltration; Net UF = UF PCt + UF Pdt.

Diabetic patients also experienced a greater decrease in urine output compared to non-diabetic, starting from a higher baseline urine output (*p* = 0.013); however, this statistical significance was lost after 12 months. The net water balance was adequate in both groups during the follow up period. The average concentration of glucose increase in the cycler is aimed at maintaining the net water balance and optimizing the hemodynamic status of the patient in addition to, controlling volume overload, edema and hypertension.

In absolute terms, daily UF is held for two years, but in terms of percentage decreases relative to the higher UF achieved with the cycler after increasing the average concentration of glucose to maintain the water balance. Urine output decreases throughout the follow-up period of two years but can still contribute about 30% to water balance.

Regarding the sodium balance (Table 7) no intergroup differences in sodium excretion over follow up period. There are intragroup differences in the removal of sodium in the PCt, which was very low at baseline and increases within time. It is offset by the peritoneal removal during the PDt, which is maintained during follow-up. In addition, the removal of sodium in the urine output decreases with loss of renal function.

**Table 7:** Sodium balance (mmol/day).

**Diabetics**	**Basal (a)**	**12 months (b)**	***p* (a *vs.* b)**	**24 months (c)**	***p* (a *vs.* c)**
TMNaPCt (1)	4.25 ± 52.20	37.02 ± 53.74	0.006	25.12 ± 59.46	0.187
TMNaPDt (2)	38.76 ± 54.51	37.39 ± 44.35	0.894	40.07 ± 50.52	0.906
TMNaPNet (3)	43.00 ± 54.81	74.41 ± 65.18	0.005	65.18 ± 78.55	0,193
Diuresis (4)	66.9 2 ± 40.38	46.05 ± 38.75	0.001	26.01 ± 25.55	0.000
Total (5)	109.93 ± 68.45	120.46 ± 72.97	0.000	91.19 ± 84.70	0.000
% PDt/P Net	90%	50.25%	-	61.48%	-
% Renal/Total	60.86%	38.23%	-	28.52%	-
**Non-Diabetics**	**Basal (a)**	**12 months (b)**	***p* (a *vs.* b)**	**24 months (c)**	***p* (a *vs.* c)**
TMNaPCt (6)	6.61 ± 64.19	32.26 ± 55.55	0.024	36.41 ± 45.15	0.049
TMNaPDt (7)	41.96 ± 32.69	40.27 ± 39.06	0.871	44.16 ± 50.78	0.851
TMNaPNet (8)	48.56 ± 73.51	72.54 ± 71.33	0.123	77.98 ± 73.53	0.149
Diuresis (9)	49.05 ± 45.51	39.35 ± 40.39	0.390	44.17 ± 49.36	0.695
Total (10)	97.61 ± 80.13	111.89 ± 87.49	0.479	121.56 ± 72.70	0.242
% PDt/P Net	86.41%	55.51%	-	56.63%	-
% Renal/Total	50.25%	35.16%	-	36.34%	-

TMNa: peritoneal sodium removal; PCt: peritoneal cycle time; PDt: peritoneal day time; TMNaP Net = TMNaPCt + TMNaPDt; P: peritoneal.

This is due to low diffusive transport secondary to the low basal UF, not causing decrease in the initial intraperitoneal sodium (132 mmol/L).The rules of low initial glucose concentration to preserve the functionality of the peritoneal membrane from the glucose toxicity in the medium to long-term, must be accompanied by emphasizing a low sodium diet.

The percentage contribution of PDt fraction removal of sodium is higher especially at the beginning of the follow-up period, and decreases over time as removal of sodium at the PCt increases (as the peritoneal UF is increased). At the end of the follow-up it still remains above 50%, while the share of RRF fraction decreased during follow-up.

Multivariate analysis was performed. In addition to intrasubjects (parameters adequacy in each time of follow up period), intersubject factors (gender, underlying disease, peritoneal transport, icodextrin and peritonitis) were taken into account. In order to evaluate the effects of both the intrasubject and intersubject factors, the interactions between them were evaluated. Additionally, we analyze the individual effects of these factors. For this purpose, we gathered patients by gender (G) (male *vs.* female), underlying disease (UD) (diabetics *vs.* non-diabetics), peritoneal transport (PT) (high + high average *vs.* low-low average), icodextrin (I) (yes *vs.* no) and peritonitis (P) (yes *vs.* no). In the table we only represent the significant values. For the peritoneal dialysis adequacy parameters, pKt/V, pCrC and pUF, the results are shown in Table 8.

**Table 8:** Multivariate analysis of adequacy dialysis parameters of peritoneal fraction, intrasubject and intersubject effects.

**Intrasubject Effects**						
	pKt/V		pCCr		pUF Net	
	F	*p*	F	*p*	F	*p*
T	1.55	0.221	0.51	0.605	5.31	0.008
**Intersubject Effects**						
G × UD × P	4.96	0.034	3.83	0.060	0.24	0.630
PT × I × P	2.10	0.158	4.34	0.046	0.04	0.842

pKt/V: peritoneal urea index, urea clearance (Kt) relative to distribution volume (V) (or total body water); pCCr: peritoneal creatinine clearance; pUF Net: peritoneal ultrafiltration; F: Snedecor F statistic; *p*: *p* value; T: time; G: gender; UD: underlying disease; PT: peritoneal transport; I: icodextin; P: peritonitis.

In the intrasubject effects, the only factor that modifies any of the parameters dialysis adequacy is the time of follow-up, in relation to the UF. The reason for this, obviously, is not the follow-up time, but the changes in the average concentration of glucose in the dialysate of the PCt, whose effect is translated into an increase in the UF.

While the intersubject effects on the peritoneal UF is not altered by any factor, peritoneal Kt/V is affected by the interaction of gender, underlying disease and peritonitis. In addition, peritoneal creatinine clearance is affected by the interaction of peritoneal transport, the icodextrin and peritonitis. Meanwhile, we should clarify that the peritoneal transport categories are performed based on PET creatinine.

For the peritoneal sodium removal (pTMNa), RRF and urine output the results are shown in Table 9.

**Table 9:** Multivariate analysis of peritoneal sodium removal net (pTMNa) Net, residual renal function (RRF) and urine output, intrasubject and intersubject effects.

**Intrasubject Effects**						
	pTMNa Net		RRF		Urine Output	
	F	*p*	F	*p*	F	*p*
T	2.74	0.073	16.09	0.000	7.66	0.001
T × UD	0.04	0.958	3.39	0.001	3.87	0.027
T × PT × P	0.25	0.777	1.68	0.194	4.26	0.019
**Intersubject Effects**						
P	4.47	0.043	2.54	0.122	2.88	0.100
I × P	3.41	0.075	5.37	0.028	2.35	0.136

TMNaP Net: peritoneal sodium removal net; F: Snedecor F statistic; *p*: *p* value T: time; G: gender; UD: underlying disease; PT: peritoneal transport; I: icodextin; P: peritonitis.

In the intrasubject effects, sodium peritoneal transport is not modified by any factor. In the intrasubject effects, regarding RRF and urine output, multivariate analysis confirmed the results of the univariate analysis. Both parameters change with the follow-up time and underlying disease. The urine output is affected by the interaction between peritoneal transport and peritonitis.

While in the intersubject effects, the sodium peritoneal transport is modified by peritonitis, the RRF is affected by interaction of icodextrin and peritonitis. The urine output is not affected by any factor.

## 4. Discussion

In this retrospective study, we compared the efficiency of automated peritoneal dialysis with similar therapy schemes using the cycler during the night time and wet day, by the use of combined solutions in DN *vs.* NDN patients. The damp day and residual renal function are determinants in the achievement of the objectives dose of dialysis, as well as in the water and sodium balance. The cause of chronic kidney disease (CKD) does not seem to influence the cleansing effectiveness of the technique.

Strategies for improving long-term survival in peritoneal dialysis patients, in general, are well defined [21]. Peritoneal dialysis (PD) is not contraindicated in end stage renal disease (ERSD) diabetic nephropathy patients, and it is a fully established form of renal replacement therapy. In addition, well-defined treatment schemes in many patients, small solute clearance and mainly slow sustained ultrafiltration (UF), can be achieved [5,22].

The ADEquacy of Peritoneal Dialysis in MEXico (ADEMEX) study changed the perception of PD prescription, as it showed that survival is less dependent on small-solute clearance, the optimizing water and salt balance as the preservation of residual renal function having more importance. However, there must be a minimum level of small-solute clearances needed to prevent uraemia related to morbidity and mortality [23]. On the other hand, the conclusions from the EAPOS (European APD Outcomes study) study, a trial performed in 177 anuric APD patients, showed that ultrafiltration (UF < 750 mL/day) is a significant predictor of mortality, whereas the creatinine peritoneal clearance does not predict survival [24].

Automated peritoneal dialysis has been used in more patients, 30.3% in developed countries [6]. Probably, in our opinion, based on the rule that an increasing small-solute clearance and therefore, a better adequate dialysis dose and control of water balance and salt, has a significant impact on the clinical course. Increased small solute clearance in PD is mainly achieved by increasing the fill volume and the number of exchange, and this is only possible with automated techniques [25,26,27].

Low glucose system therapy, using solutions in combination, has been designed to help patients reduce glucose load and exposure, to decrease the risk of developing co-morbidities associated with elevated glucose, in addition to minimizing the toxic effects of glucose in the peritoneal membrane. On the one hand, amino acid containing solutions have been shown to improve glucose and lipid metabolism [28]. On the other hand, a randomized controlled trial in Mexican diabetic patients demonstrated to improve the control of multiple metabolic variables with icodextrin. Moreover, it suggests that it provides greater fluid removal and small solute clearance and does not cause any damage to residual renal function [29,30].

A recent meta-analysis showed that icodextrin prescription improved peritoneal ultrafiltration and mitigated uncontrolled fluid overload. There were no significant effects on peritonitis, on the technique, patient survival and RRF. No harm was identified with their use. Based on the best available evidence, the use of these “biocompatible” PD solutions, lead to clinically relevant benefits without adding risk of harm [31].

Results and conclusions from the combined (IMPENDIA/EDEN) trials (The Improved Metabolic Control of Physioneal, Extraneal, Nutrineal (P-E-N) *versus* Dianeal Only in DIAbetic continuous ambulatory peritoneal dialysis (CAPD) and automated peritoneal dialysis (APD) Patients (IMPENDIA), and The Evaluation of Dianeal, Extraneal, Nutrineal (D-E-N) *versus* Dianeal only in Diabetic CAPD Patients (EDEN)) showed that a low-glucose PD regime improves metabolic indexes in diabetic patients receiving peritoneal dialysis, but may be associated with an increased risk of extracellular fluid volume expansion. Thus, the use of glucose-sparing regimens in peritoneal dialysis patients should be accompanied by close monitoring of fluid volume status [7,9].

From this point view, a low-glucose prescription should always be considered when managing diabetic patients on peritoneal dialysis.

Residual renal function (RRF) is of paramount importance in patients with end-stage renal disease, with benefits that go beyond contributing to achieve the adequacy targets [10]. Two recent papers review the strategies for preserving RRF in peritoneal dialysis patients [13,14]. Since the reanalysis of the CANUSA data [32], it has been acknowledged that there is an important association between RRF and survival in patients on peritoneal dialysis. This association is stronger when RRF is expressed as asurinary output rather than as small solute clearance, suggesting that preservation of a good hydration balance is more important than clearance [33,34].

McCafferty and colleagues reported on a retrospective analysis in peritoneal dialysis patients, in whom fluid status was assessed by multifrequency bioimpedance, that increments and decrements in ECW/TBW were not associated with preservation or reduction in RRF and there was no significant correlation seen between change in hydration status and subsequent loss in RRF. Their study did not support the view that overhydration preserves residual renal function and above all the risks to persistent hypervolemia [35].

Rodriguez-Carmona *et al.* have shown that standard APD schedules are frequently associated with poor Na removal rates and this may influence the cardiovascular outcomes in APD patients [36].This low sodium removal is justified, due to the short dwell schedule during the night session that may result in significant Na sieving and less efficient Na removal of this cation [37]. They have also suggested that longer nocturnal dwell times and supplementary diurnal exchanges, can improve Na removal in APD [30]. Fourtounas *et al.* reported that when icodextrin is used for the long dwell as an adjuvant for higher daily UF, this resulted not only in increasing solute clearance but also in removing more sodium [38].

This study does not attempt to analyze the metabolic control or the survival of patients, just to assess the results of residual renal function and the similar schemes in automated peritoneal dialysis, such as clearance of solutes, water and salt balance in diabetic and non-diabetic patients; during a follow-up period of 24 months and try to determine what factors may modify them. We did not find references where authors compared their results in both populations of APD patients.

In our Home Therapies Unit, when the schema dialysis for a patient starting renal replacement therapy, DPA is planned. In our mind there are three objectives: (1) to preserve renal function; (2) preserving the functionality of the peritoneal membrane and (3) dialysis adequacy, control water and sodium balance.

In the dialysis planning, we take into account factors that, in our opinion, can influence the dynamics of peritoneal transport: (1) consider non-modifiable factors such as age, diabetes status, comorbidity, and intrinsic permeability of the peritoneal membrane; (2) consider modifiable factors, like inflammation, nutritional status, emotional status (e.g., depression *vs.* no depression), and the prescription-related factors (biocompatibility of the solutions, combined used solutions, solute transport, sodium excretion and ultrafiltration); and (3) random factors, peritonitis and others. We considered them as a whole, by matching multiple possibilities in the individualization therapy.

In this analysis process, certain cycler parameters were unchanged, (total time, total volume, volume infusion) as well as the two daily period exchanges. Therefore, in relation to the parameters which quantify the dose of dialysis: (1) with respect to the index Kt/V changes observed are justified by changes in the volume distribution (V = TBW), which was determined by anthropometric equations for men and women where weight is a critical factor. As patients monitor themselves, intrasubject variability is determined by changes in weight. Therefore, in stable conditions the equations can be used in clinical routine to determine the TBW, taking into account the observation that there are differences in body composition between DN and NDN, and also gender. However, we cannot determine if the changes in weight, and therefore the TBW, are due to increase in lean body mass, fat body mass, or hyperhydration (without edema or increased blood pressure), or the sum all of these causes. From this point of view, to make a better assessment of these changes it is necessary to measure them with adequate equipment to determine body composition and hydration status, either with single frequency bioimpedance vector (BIVA) or multiple frequency bioimpedance spectroscopy (BIS) [35,39,40,41,42]; (2) With regards to creatinine clearance, no changes in net peritoneal clearances or their fractions of nocturnal and diurnal periods were found. Decreasing the overall clearance is due to residual renal function loss.

Regarding the Kt/V index and creatinine clearance, there were no determinant factors in the intrasubject effects, either separately or by matching them. However, interaction is observed in the intersubject factors matching. On the one hand, in the pKt/V (gender, disease and peritonitis) and in the pCrCl (peritoneal transport, icodextrin and peritonitis), it is suggested, that while peritonitis by itself has no intrasubject and intersubject effects, interaction with other factors can result in significant changes in the estimation of dialysis adequacy parameters.

The daytime dialysis fraction of both parameters provides sustained benefit in the follow-up time, with an average of 30% on pKt/V and 40% on the pCrCl in both DN and NDN. Renal fraction decreases over time, but still provides a yearly average of 20% and 40% respectively. Therefore, the rule of RRF preservation and maintenance is necessary to achieve the objectives.

The water and sodium balance go together. With time, the glucose concentration in the night period is increased to augment water elimination therefore, optimizing the hemodynamic status of the patient, control volume overload, edema and hypertension. In a dialysis machine, the concentration of glucose in each cycle varies according to the ultrafiltration profile we have established [43], and water removal as sodium removal also increases.

Although profiles have been established, the free water transport in each cycle helps to decrease the sodium concentration in the dialysate. Therefore, the sodium dip is higher and allows the diffusive transport. Therefore, as each cycle increases or decreases the glucose concentration, according to the established profile, it also rises or reduces the ultrafiltration and sodium removal.

The UF profile set will have its effects on solute clearance, the balance of water and sodium, so the descendent profile may be the best [43]. The rule of low glucose concentration at the beginning to preserve the functionality of the peritoneal membrane, must be accompanied by emphasizing a low sodium diet.

In multivariate analysis, in the intrasubject effects, we did not observe effects of factors alone or in combination on the net peritoneal transport of sodium. The follow-up time and the interaction with the underlying disease is crucial to the RRF and urine output. Keep in mind that diabetic patients initially started with a better RRF and more urine output compared to non-diabetic.

The urine output objective is the interaction between the peritoneal transport and peritonitis. Intersubject effects were not observed on the urine output. However, a direct peritonitis effect on peritoneal net sodium balance is observed. With respect to the RRF, we observed interaction between icodextrin and peritonitis factors. Peritonitis theoretically can help to reduce RRF either directly, as in severe infection, or indirectly by nephrotoxic antibiotic use.

The loss of the slope of RRF is more pronounced in DN than in NDN patients, especially at baseline time interval to 12 months (loss of 0.292 mL/month *vs.* 0.132 mL/month respectively), and is attenuated in the range from 12 to 24 months (loss of 0.135 mL/ month *vs.* 0.097 mL/month respectively).

As with the parameters of dialysis adequacy in water and sodium balance, the daytime period has sustained benefits in follow-up time, representing over 30% in UF, and over 50% in sodium balance. At baseline study, it has a greater importance due to use of low glucose concentration in the cycler. The fraction in renal sodium and water removal decreases over a period of 24 months, but still provides an average profit of approximately 30% for both parameters.

This study suffers from the general limitations of observational studies, the small number of patients included in each group, and results should thus be interpreted with caution.

## 5. Conclusions

In conclusion, in the present observational study, automated peritoneal dialysis combines multiple possibilities in the individualization of therapy. It is effective in providing dialysis dose and adequate water and sodium balance when efficient schemes therapy and solutions combination are prescribed. The diurnal period has an important value achieving objectives in both DN and NDN patients.
